# Exon identity crisis: disease-causing mutations that disrupt the splicing code

**DOI:** 10.1186/gb4150

**Published:** 2014-01-23

**Authors:** Timothy Sterne-Weiler, Jeremy R Sanford

**Affiliations:** 1 Banting and Best Department of Medical Research, University of Toronto, 160 College St, Toronto, ON M5S 3E1, Canada; 2 Department of Molecular, Cellular and Developmental Biology, University of California Santa Cruz, 1156 High Street, Santa Cruz, CA 95060, USA

## Abstract

*Cis*-acting RNA elements control the accurate expression of human multi-exon protein coding genes. Single nucleotide variants altering the fidelity of this regulatory code and, consequently, pre-mRNA splicing are expected to contribute to the etiology of numerous human diseases.

## Introduction

Although genes span 33.4% of the human genome from start codon to stop codon, only 3.66% of their sequence comprises protein coding sequences [1]. Introns make up the rest of this gene space, separating adjacent protein coding exons from one another. To produce a mature mRNA that encodes a continuous string of codons, these exons must be put together following the precise excision of introns in a process referred to as precursor messenger RNA splicing (pre-mRNA splicing). Aberrant pre-mRNA splicing is now recognized as the underlying cause of many human diseases. Mutations in *trans*-acting factors or *cis*-acting regulatory elements compromise the expression of protein-coding genes by decreasing the specificity or fidelity of splice site selection, a fundamental step in expression of multi-exon genes.

At least three mechanisms can induce RNA-based disease. First, genetic variants such as point mutations can abolish *cis*-acting elements recognized by RNA binding proteins (RBPs), thereby inducing disease phenotypes in humans. Work on many disease genes, including the breast cancer gene *BRCA1*, the gene encoding the cystic fibrosis transmembrane conductance regulator (*CFTR*), the growth hormone gene *GH1* and the ataxia telangiectasia mutated gene *ATM*, has demonstrated that all classes of point mutations, including nonsense mutations, can disrupt exonic splicing regulatory elements (ESRs) and induce aberrant pre-mRNA splicing [2-6]. Second, RBPs are implicated (either by mutation or aberrant expression) in numerous human diseases, including cancer, Alzheimer’s disease, frontotemporal dementia, spinal muscular atrophy (SMA) and retinitis pigmentosa (reviewed in [7]). These observations suggest that processing of specific transcripts or, more likely, families of related transcripts may be mis-regulated. Finally, RNAs transcribed from genes containing trinucleotide repeat expansions also induce disease. These toxic RNAs seem to function by sequestering RBPs and causing gross changes in post-transcriptional gene expression programs [8].

Given that other review articles have already done an exceptional job at summarizing the pleiotropic effects of RBPs and toxic RNA elements on pathogenesis [9-13], here we focus on aberrant protein-RNA interactions implicated in monogenic human diseases.

## Splicing mechanism

**Figure 1:**
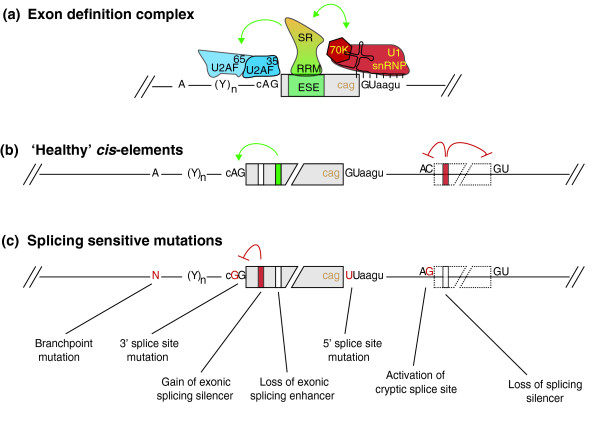
**Targets of single-nucleotide polymorphism-induced aberrant pre-mRNA splicing mutations. (a)** Schematic diagram of the exon definition complex. 70 K, 70 kDa subunit of the U1 snRNP; ESE, exon splicing enhancer; RRM, RNA recognition motif protein domain; SR, serine-arginine rich protein domain; U1 snRNP, U1 small nuclear ribonucleoprotein; U2AF, U2 auxiliary factor; Y, pyrimidine nucleotide. Uppercase indicates donor and acceptor splice site dinucleotides, lowercase indicates adjacent consensus nucleotides. **(b)** Typical functionality of splicing regulatory elements in wild type (healthy) context. **(c)** Potential mechanisms for splicing-sensitive mutations. Green squares and arrows indicate splicing enhancers; red indicates silencers; solid boxes indicate constitutive or alternative exons; dashed boxes indicate pseudo-exons.

Pre-mRNA splicing is catalyzed by the spliceosome, a large ribonucleoprotein complex. The spliceosome assembles *de novo* on each and every transcribed intron and catalyzes two sequential *trans*-esterification reactions, which yield ligated exons and an excised intron-lariat [14]. The earliest stages of spliceosome assembly are critical in defining which exon sequences are to be joined during the splicing reaction [15]. Splicing of most human pre-mRNAs initiates in an exon-centric manner in which the upstream 3′ splice site and the downstream 5′ splice site are linked through interactions between U2 auxiliary factor (U2AF) and the U1 small ribonucleoprotein particle (U1 snRNP; Figure 1a) [16]. Later in the spliceosome assembly pathway these cross-exon interactions are replaced by intron-bridging interactions that connect the reactive 5′ and 3′ splice sites [16,17].

The decision of whether to splice or not to splice is typically modeled as a stochastic rather than deterministic process, such that even the most defined splicing signals can sometimes splice incorrectly [18]. However, under normal conditions, pre-mRNA splicing proceeds at surprisingly high fidelity [19]. This is attributed in part to the activity of adjacent *cis*-acting auxiliary exonic and intronic splicing regulatory elements (ESRs or ISRs) [20-24]. Typically, these functional elements are classified as either exonic or intronic splicing enhancers (ESEs or ISEs) or silencers (ESSs or ISSs) based on their ability to stimulate or inhibit splicing, respectively. Although there is now evidence that some auxiliary *cis*-acting elements may act by influencing the kinetics of spliceosome assembly, such as the arrangement of the complex between U1 snRNP and the 5′ splice site, it seems very likely that many elements function in concert with *trans*-acting RBPs [25]. For example, the serine- and arginine-rich family of RBPs (SR proteins) are a conserved family of proteins [26] that have a key role in defining exons [27]. SR proteins promote exon recognition by recruiting components of the pre-spliceosome to adjacent splice sites or by antagonizing the effects of ESSs in the vicinity [28-30]. The repressive effects of ESSs can be mediated by members of the heterogeneous nuclear ribonucleoprotein (hnRNP) family and can alter recruitment of core splicing factors to adjacent splice sites [31]. In addition to their roles in splicing regulation, silencer elements are suggested to have a role in repression of pseudo-exons, sets of decoy intronic splice sites with the typical spacing of an exon but without a functional open reading frame [32]. ESEs and ESSs, in cooperation with their cognate *trans*-acting RBPs, represent important components in a set of splicing controls that specify how, where and when mRNAs are assembled from their precursors [30,33,34].

## Alternative splicing

The sequences marking the exon-intron boundaries are degenerate signals of varying strengths that occur at high frequency within human genes [35]. In multi-exon genes, different pairs of splice sites can be linked together in many different combinations, creating a diverse array of transcripts from a single gene [36,37]. This is commonly referred to as alternative pre-mRNA splicing, and is classified into several discrete event types that have been observed both *in vitro* and *in vivo*[38].

Recent studies suggest that 86 to 94% of human multi-exon genes undergo alternative splicing [39,40] and a considerable portion of human functional variation within the population is likely to cause changes at the transcript level [41]. The sheer abundance of this phenomenon is remarkable, raising the question of how many of the isoforms produced by a single gene encode functional messages. Although most mRNA isoforms produced by alternative splicing will be exported from the nucleus and translated into functional polypeptides, different mRNA isoforms from a single gene can vary greatly in their translation efficiency [42]. Those mRNA isoforms with premature termination codons at least 50 bp upstream of an exon junction complex are likely to be targeted for degradation by the nonsense-mediated mRNA decay (NMD) pathway [43]. Although this type of unproductive splicing is typically thought to be a byproduct of splicing as a stochastic process, the SR genes are clear examples of how this can be exploited as an essential regulatory mechanism [44-46]. SR proteins have been shown to regulate the splicing of their own genes, each of which contain an ultraconserved sequence [47] such as a poison exon containing a premature termination codon; when spliced into the mature RNA, these exons can trigger transcript degradation by NMD [48,49]. The first example of this form of splicing factor autoregulation coupled to mRNA surveillance was characterized in the *SRSF2*/*SC35* gene (a member of the SR family): high levels of the SRSF2/SC35 protein promote a 3′ untranslated region splicing event that destabilizes the SRSF2/SC35 mRNA [46].

## Mis-splicing and monogenic diseases

Given that exon-intron boundaries can occur at any of the three positions of a codon, it is clear that only a subset of alternative splicing events can maintain the canonical open reading frame. For example, only exons that are evenly divisible by 3 can be skipped or included in the mRNA without any alteration of reading frame. Splicing events that do not have compatible phases will induce a frame-shift. Unless reversed by downstream events, frame-shifts will almost certainly lead to one or more premature termination codons, probably resulting in subsequent degradation by NMD. The most common frame-preserving alternative event type is compatibly phased exon skipping; however, 20% of all frame-preserving alternative splicing events involve the alternative use of adjacent 3′ NAGNAG splice sites [50,51]. Several studies have investigated the evolution of multi-exon gene architectures and found significant correlation of the edges of exons with protein domain boundaries [52,53]. Furthermore, exons whose edges correlated with protein domain boundaries were significantly enriched for compatible splice site phase. These observations have been used as evidence for the evolutionary hypothesis of exon shuffling, a mechanism for diversification of modular protein functions [54,55]. Moreover, the data clearly support the postulate that evolutionary history of a gene will affect its susceptibility to alternative splicing-induced frame-shifting.

Following a model of neutral genetic drift, some genes are under greater selective constraints than others. Genes encoding proteins that have vital and non-redundant roles may impart a major loss of fitness to an organism if disrupted by germline and somatic mutation. Depending on protein structure and function and exon-intron architecture, these genes may be more or less susceptible to aberrant function by different means. For example, different mutations causing loss of function in *CFTR* can cause varying levels of severity of cystic fibrosis (CF) [56]. Although 70% of CF cases are at least heterozygous for a deletion of phenylalanine 508 (ΔF508) that impairs protein folding and subsequent function [57], only four other mutations (G542X, N1303K, G551D and W1282X) have allele frequencies above 1% [58]. This leaves a percentage of atypical CF-associated mutations that are rare or unique to individuals or families, resulting in roughly 15% of all CF cases having mutations with unknown functions [59]. Moreover, about 13 to 20% of all the CF-associated mutations are thought to cause pre-mRNA splicing defects by aberrant inclusion or exclusion of several of the 27 exons as a primary mechanism of disease causation [10]. At least one of these, exon 9, has been studied in great detail, illuminating a complex set of regulatory elements that regulate its alternative splicing [60-62].

High-throughput DNA sequencing is now revealing the extent of human genetic variation on a comprehensive scale. However, because of the complexity of these data, it is often unclear which variants are functional and which biochemical mechanisms they affect [63]. For genes that are highly susceptible to aberrant splicing by a number of different mechanisms (such as *CFTR*; Figure 1), determining the penetrance associated with *de novo* atypical mutations is a crucial gap towards comprehensive molecular diagnosis for their associated diseases. To tackle this problem for *CFTR* and other genes with pre-mRNA splicing defects, it is necessary to consider the possible mechanistic impacts of a point mutation on the splicing machinery. Figure 1b,c illustrates some of the architectural features of a generic wild-type (healthy) gene, such as: the presence of one or more exonic splicing enhancers; splicing silencers that work to repress intronic pseudo-exons; and cryptic splice sites. Mutation of 5′ and 3′ splice site dinucleotides and adjacent bases can render them inactive; this is the most easily recognized mechanism of splicing disruption, accounting for 10% of all human inherited disease mutations [64]. For this reason, disruption of the GU and AG splice site dinucleotides are recognized as deleterious by most of the recent single-nucleotide polymorphism functional classification tools, such as those based on SIFT [65,66]. However, the need for methods or tools to evaluate the impact of genetic variants towards the loss or gain of both ISRs and ESRs remains critical.

Work from our group and another suggest that 22 to 25% of exonic human inherited disease mutations are likely to be splicing sensitive [67,68]. We find that this percentage is unevenly distributed across different diseases (Tables 1 and 2), suggesting that there is a spectrum of susceptibility towards aberrant gene regulation through loss or gain of ESRs. In the case of the Duchenne muscular dystrophy (*DMD*) gene, for example, rather than the approximately 22 to 25% presented previously [67,68], we find that as many as 120 mutations, representing nearly half of all the missense and nonsense disease-causing mutations targeting this gene, cause the loss or gain of disease enriched ESRs. This suggests an expanded role for splicing mutations relating to Duchenne or the less severe Becker muscular dystrophy. Future studies that include mutations affecting intronic *cis*-acting elements may shed light on an additional class of splicing-sensitive variants.

**Table 1:** Genes sorted by percentage of ESR loss or gain mutations per gene (for genes with more than 10 such mutations)

**Gene**	**Number of ESR**	**Percentage of**
	**loss/gain mutations**	**mutations in gene**
*CEP290*	18	62.1
*CHM*	14	56.0
*FGA*	11	52.4
*AGL*	12	52.2
***PAX3***	12	50.0
*LAMB3*	13	48.1
***BRAF***	11	47.8
***NF2***	35	47.3
*NIPBL*	22	46.8
*DMD*	120	45.8
*EFNB1*	12	44.4
*KRIT1*	16	44.4
*CYP27A1*	11	44.0
*COL4A5*	84	43.5
***EXT1***	18	42.9
***TSC1***	17	42.5
*ALB*	21	42.0
*EDA*	12	41.4
*IL2RG*	28	41.2
***APC***	32	41.0
*F10*	15	40.5
***MSH2***	51	40.5
*CYBB*	53	40.2
*FECH*	12	40.0
***NF1***	89	39.6
***BRCA2***	35	38.9

The table shows genes with mutations that cause the loss or gain of a disease-enriched ESR, based on data from [68]. Cancer-associated tumor suppressor genes from the Cancer Census [69] and oncogenes are in bold.

**Table 2:** Genes sorted by total number of ESR gain or loss mutations

**Gene**	**No of ESR gains**	**Percentage of**
	**and losses**	**gene**
*F8*	221	28.4
*CFTR*	173	30.1
*LDLR*	143	27.1
*FBN1*	135	24.9
*DMD*	120	45.8
*F9*	101	32.8
***NF1***	89	39.6
*COL4A5*	84	43.5
*PAH*	79	23.0
*GLA*	69	34.8
*OTC*	66	28.9
*BTK*	64	25.7
***BRCA1***	61	35.9
*ATP7B*	60	25.5
***ATM***	58	34.3
***MEN1***	58	37.2
*ABCA4*	56	19.0
***MLH1***	55	35.7
*COL7A1*	53	28.3
*CYBB*	53	40.2
***MSH2***	51	40.5
***COL1A1***	49	21.1
*GCK*	48	28.2
***TSC2***	47	30.3
*COL1A2*	45	26.0
*MYH7*	45	23.9

The table shows genes with mutations that cause the loss or gain of a disease-enriched ESR, based on data from [68]. Cancer-associated tumor suppressor genes from the Cancer Census [69] and oncogenes are in bold.

**Figure 2:**
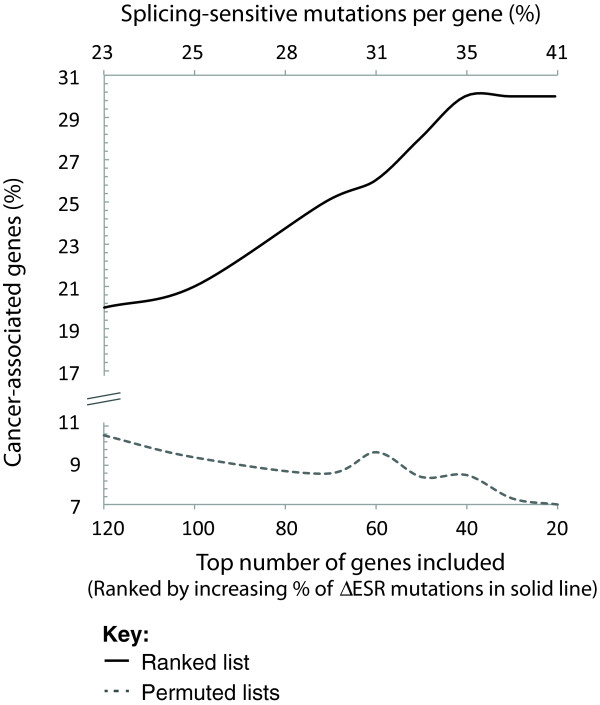
**Splicing susceptibility of cancer related genes.** The positive increase in cancer-associated genes is dependent on the overall percentage of putative splicing mutations within the gene (black line). Independent permutations followed by re-plotting without any putative splicing sensitivity measure results in expected numbers of cancer-associated genes (dotted gray line). Cancer-associated genes are defined as the 510 genes represented in the Cancer Census [69].

We find it intriguing that the architecture of different genes renders some more sensitive to mutation-induced aberrant splicing than others. Within this subset of splicing-sensitive genes we noted that the proportion of cancer-related genes [68] increases with the percentage of putative splicing-sensitive mutations (Figure 2). Of the 492 genes from the Human Gene Mutation Database considered by our analysis, only 11% are cancer related [69] while this proportion is three-fold higher within the top 50 splicing-sensitive genes (χ2 goodness-of-fit *P*-value of 1.2 × 10^-4^). Summarizing the data in Tables 1 and 2, it becomes apparent that the majority of these are known tumor suppressor genes, providing support for the motion to recognize aberrant splicing as a hallmark of cancer [70]. Aberrant splicing has already been directly implicated as a causative mechanism for disruption of many of these genes previously [71]. Missense and nonsense mutations in the mismatch repair genes *MLH1* and *MSH2* have both been shown to cause aberrant splicing in multiple contexts [72-75]. Likewise, mutations in the APC tumor suppressor gene have aberrant isoforms that are thought to be degraded by NMD [76]. These data suggest the intriguing hypothesis that cancer-related genes may have a greater susceptibility towards aberrant splicing than other genes.

## Future therapeutic potential

The susceptibility of many disease genes (such as *DMD*, *ATM*, *NPC1* (Niemann-Pick disease), *F9* (hemophilia A), *F8* (hemophilia B)) to aberrant pre-mRNA splicing has spawned creative therapeutic approaches that have been the focus of a great deal of time and effort [77-84]. Of these, one of the most successful cases has been the reversal of aberrant exon 7 skipping in the SMA-related gene *SMN2* by antisense oligonucleotides [79,83,84]. SMA is an autosomal recessive disorder that is characterized by varying severity due to the loss of function of *SMN1*, of which humans have one copy on each chromosome 5. A nearly identical paralog, *SMN2*, has only five single nucleotide differences, all of which are non-coding except one C > T synonymous mutation six bases from the 3′ splice site within exon 7. The mechanistic impact of this C > T transition has been studied extensively, and has been shown to be associated with both the loss of an ESE that binds SRFS1 to stimulate exon definition [85,86] and the antagonistic gain of an ESS that binds hnRNP A1 to repress exon definition [5]. *In vivo* selection studies and antisense oligonucleotide tiling experiments have additionally discovered a number of other regulatory elements within and adjacent to this exon [80,87,88].

Because individuals with SMA typically have loss of *SMN1* but normal copies of *SMN2*, research into a general treatment for SMA has been targeted towards methods to increase splicing of endogenous *SMN2* exon 7 as a means to increase functional SMN protein. Recent studies have robustly ameliorated symptoms of severe SMA mouse models through delivery of antisense oligonucleotides masking the ESS-N1 element [79,83,84], demonstrating that antisense approaches may represent an effective treatment for SMA. Although most inherited disease-related genes do not have a backup copy similar to *SMN2* to serve as a template for RNA targeted therapies, this scenario does illuminate the potential feasibility of rational nucleic acid-based therapeutics in the coming years.

## Conclusion

Functional characterization of both germline and somatic variants remains a considerable challenge. This is due in part to the limited understanding of the gene architectural contexts that give rise to varying degrees of susceptibility to aberrant processing. How different degrees of susceptibility contribute to the etiology of inherited and somatic diseases remains a crucial question in the field. This question is becoming increasingly important for several inherited and somatic diseases, including cancer. The root of this question lies at the heart of unraveling the networks of protein-RNA interaction that are active in various cellular contexts. Beyond this task lies the promise of potentially groundbreaking therapeutic approaches based on correcting aberrant protein-RNA interactions within a cell.

## Abbreviations

CF: Cystic fibrosis; CFTR: Cystic fibrosis transmembrane conductance regulator; ESE: Exonic splicing enhancer; ESR: Exonic splicing regulator; ESS: Exonic splicing silencer; ISE: Intronic splicing enhancer; ISS: Intronic splicing silencer; NMD: Nonsense-mediated decay; RBP: RNA binding protein; SMA: Spinal muscular atrophy; snRNP: small ribonucleoprotein particle; SR: Protein serine- and arginine-rich protein.

## Competing interests

The authors declare that they have no competing interests.
